# A serendipitous journey to a promoter variant: The c.‐106C>A variant and its role in late‐onset ornithine transcarbamylase deficiency

**DOI:** 10.1002/jmd2.12289

**Published:** 2022-04-12

**Authors:** Ashley Hertzog, Arthavan Selvanathan, Rebecca Halligan, Timothy Fazio, Gerard de Jong, Drago Bratkovic, Kaustuv Bhattacharya, Adviye Ayper Tolun, Bruce Bennetts, Katrina Fisk

**Affiliations:** ^1^ NSW Biochemical Genetics Service The Children's Hospital at Westmead Westmead New South Wales Australia; ^2^ Genetic Metabolic Disorders Service The Children's Hospital at Westmead Westmead New South Wales Australia; ^3^ Metabolic Unit Women's and Children's Hospital Adelaide South Australia Australia; ^4^ Metabolic Diseases Unit Royal Melbourne Hospital Parkville Victoria Australia; ^5^ Melbourne Medical School University of Melbourne Parkville Victoria Australia; ^6^ Disciplines of Genomic Medicine and Child and Adolescent Health Faculty of Medicine and Health, University of Sydney Sydney New South Wales Australia; ^7^ Department of Molecular Genetics Western Sydney Genetics Program, The Children's Hospital at Westmead Westmead New South Wales Australia

**Keywords:** inborn errors of metabolism, ornithine transcarbamylase deficiency, promoter variants, urea cycle disorder

## Abstract

Ornithine transcarbamylase deficiency (OTCD) is an X‐linked urea cycle disorder characterised by reduced or absent OTC enzyme activity, resulting in the accumulation of neurotoxic ammonia. Approximately 80%–90% of the causative variants are identified by Sanger sequencing or multiplex ligation‐dependent probe amplification (MLPA) of the *OTC* gene. A 23‐year‐old male with biochemical evidence of OTCD was referred for molecular analysis. Initial Sanger sequencing yielded no pathogenic variants. MLPA testing raised suspicion of a mosaic deletion of exon 1; however, high‐resolution microarray did not identify a copy number variant on the X chromosome. Sequencing over the suspected breakpoint detected a hemizygous likely pathogenic promoter variant, c.‐106C > A, which was located within the MLPA probe binding site. Subsequently, historical patients referred to our centre, without a molecular aetiology for their OTCD, were re‐sequenced with these primers and this variant was also identified in two additional unrelated males. All three patients described in this case series have the late‐onset disease. Two presented at 5 years of age with vomiting, whilst the other was managed from birth based on a family history of late‐onset OTCD. One patient required liver transplantation due to recurrent decompensations; the other two are managed with a protein‐restricted diet. All three patients have not sustained any significant neurological insults and are functioning well as adults. These cases support screening of the promoter region within the *OTC* gene, particularly if a molecular basis has not been elucidated by MLPA or sequencing of the coding regions.

## 1 INTRODUCTION

Ornithine transcarbamylase deficiency (OTCD) (OMIM # 311250) is an X‐linked condition that accounts for at least half of all inherited urea cycle disorders. Ornithine transcarbamylase (OTC) (EC 2.1.3.3), encoded by the *OTC* gene located on the short arm of the X chromosome (Xp11.4), is essential in converting neurotoxic ammonia into urea in the liver. Deficiency of this enzyme results in a build‐up of ammonia in the bloodstream.^1^ Males often present in the newborn period (within 4 weeks) with symptomatic hyperammonaemia, manifesting as poor feeding and lethargy, progressing to coma and even death, if untreated. However, late‐onset presentations (ranging from infancy to adulthood) can have varying clinical features, including developmental delay, psychosis and encephalopathy, usually precipitated by a protein load, intercurrent illness or medication.^2^ Although commonly asymptomatic, heterozygous females may also manifest variable diseases. Females often experience an attenuated clinical course and are less likely to progress to coma and death, although severe adverse outcomes can occur.^3^, ^4^

The diagnosis of OTCD is made based on characteristic biochemical findings: hyperammonaemia, raised glutamine, low citrulline and arginine in plasma, as well as orotic aciduria. Routine diagnostic molecular analysis (Sanger sequencing of the coding region and exon/intron boundaries plus deletion/duplication analysis) is often requested for confirmatory and reproductive purposes, and identifies the aetiology of 80%–90% of cases.^5^ While it is possible to identify large *OTC* deletions in males due to a lack of polymerase chain reaction (PCR) amplification, multiplex ligation‐dependent probe amplification (MLPA) analysis is often required for the detection of duplications and mosaic deletions in males.^6^ There are case reports of individuals with biochemically‐confirmed OTCD, where no pathogenic variants were identified during routine screening. However, the further analysis identified deep intronic, promoter or enhancer variants within the *OTC* gene.^7^, ^8^, ^9^

We report three additional cases of late‐onset OTCD caused by a promoter variant in the *OTC* gene; c.‐106C>A (NC_000023.10:g.38211844C>A). There was a substantial delay in ascertaining their molecular aetiology, as this variant was not clearly identifiable by routine molecular analysis. All three patients have had a generally favourable clinical outcome with appropriate treatment. We discuss their clinical histories in detail and highlight the importance of including interrogation of the promoter region of *OTC* during routine molecular analysis.

## 2 PATIENT 1

Patient 1 was born via Caesarean section at 29 weeks gestation due to maternal HELLP (haemolysis, elevated liver enzymes and low platelet count) syndrome. He had a male half‐sibling, with shared maternal inheritance, who developed persist vomiting after a large protein meal. This sibling was diagnosed biochemically with OTC deficiency at 12 years of age and died aged 33 years with hyperammonemic encephalopathy shortly after an overseas holiday. He had been intellectually normal, achieving a senior management role.

Patient 1 was presumed to have OTCD and was managed with a low protein diet from birth. Allopurinol load test at 6 weeks of age showed normal urinary orotate (10 mmol/mol creatinine: normal range < 15.5) and elevated orotidine (25 mmol/mol creatinine: normal range <7). Despite this, his dietary restriction and arginine supplementation were maintained during childhood. He had multiple intercurrent illnesses with no documented hyperammonaemia. He had an IQ of 64 at 8 years, but completed high school education at mainstream school, without learning support, proceeding to vocational education. He currently lives with his parents and is employed as a sales assistant.

As an adult, his protein intake has been liberalised and he has been asymptomatic after consuming high‐protein meals. He came to the attention of the metabolic service, wanting molecular confirmation of OTCD. He had a normal physical examination, including normal growth parameters, with no signs of chronic liver disease.

Biochemical investigations revealed normal fasting plasma ornithine, arginine and citrulline concentrations with mildly elevated glutamine (760 μmol/L: normal range 420–700). Ammonia and urinary orotate concentrations were normal. Repeat allopurinol load test demonstrated an elevated urinary orotate (27.3 mmol/mmol creatinine: normal range 0–4.9) consistent with a urea cycle defect. Microarray analysis showed an interstitial hemizygous deletion of ~0.5 megabases at Xq21.1, unrelated to the phenotype. The chromosome region Xp11.4 containing the *OTC* gene appeared normal. Routine Sanger sequencing of the *OTC* gene, in 2017, failed to identify a pathogenic variant.

## 3 PATIENT 2

Patient 2 presented as a five‐year‐old boy, with several weeks of persistent vomiting. He was born via elective caesarean section at term, with no complications. He had a background of mild expressive speech delay, attributed to hearing loss secondary to otitis media with effusion. He self‐selected a low protein diet and his mother also preferred a vegetarian diet.

Urine organic acid analysis showed an elevation in orotate. He was subsequently found to have raised ammonia and glutamine, although the exact values are no longer available. Allopurinol load test showed increased urinary orotate (22 μmol/mmol creatinine (pre) to 51 μmol/mmol creatinine (post): normal range <7). Ammonia was elevated (98 μmol/L: normal range 20–80) post allopurinol challenge. He was commenced on sodium phenylbutyrate and arginine, and advised to continue with self‐limitation of protein intake with avoidance of large amounts of meat, cheese and milk.

Liver biopsy at six years of age showed focal portal chronic inflammation and interface hepatitis, with prominent nuclear glycogenation. The liver enzyme activity of OTC was reduced (110 nmol/min/mg protein: normal range > 200). Routine Sanger sequencing of the *OTC* gene, in 2006, failed to identify a pathogenic variant. Subsequent next‐generation sequencing (a urea cycle disorders panel) in 2013 was also negative for any known pathogenic variants.

Patient 2 completed mainstream schooling and is now working as an apprentice in a trade. He remains on sodium phenylbutyrate, sodium benzoate and arginine, and limits natural protein intake to around 0.8 g/kg/day.

Interestingly, the older brother of Patient 2 had a developmental delay in childhood and also self‐selected a low protein diet. At age 10, he had a prolonged generalised tonic–clonic seizure during an intercurrent illness, with no cause identified. He also had an abnormal allopurinol load test and reduced liver OTC enzyme activity (67 nmol/min/mg protein: normal range > 200), but molecular testing was not performed.

## 4 PATIENT 3

Patient 3 presented as a five‐year‐old boy with an 18‐month history of unusual behaviours, with intermittent vomiting and lethargy. There was no significant antenatal or perinatal history. His mother had a history of migraines.

Historical records indicated his ammonia levels were mildly elevated (exact values unavailable). Urine metabolic screen demonstrated an increase in orotate (33 μmol/mmol creatinine: normal range < 10). Plasma glutamine was elevated (1195 μmol/L: normal range < 907 μmol/L), and plasma arginine and citrulline were within the normal range. The mother and maternal grandmother underwent protein and allopurinol load tests. They remained asymptomatic, but demonstrated increased orotate excretion.

Patient 3 was commenced on a protein restriction of 1.75 g/kg, which was gradually reduced to 1.1 g/kg. He was prescribed sodium benzoate, sodium phenylacetate and arginine and was noted to have increased energy levels after commencement. However, he had multiple admissions to his local hospital each year (four to five) with ammonia concentrations increasing to 200–350 μmol/L.

A formal developmental assessment at age seven using the WISC‐III (Wechsler Intelligence Scale for Children—Third Edition) tool demonstrated good general abilities (58th centile), with specific weakness in reading, arithmetic and attention. It was apparent that even in between admissions, the patient's cognitive state was not normal, and there was limited progress even with the addition of sodium phenylbutyrate. There were ongoing admissions (87 admissions prior to age 17) and prolonged periods of encephalopathy outside of hospital, despite the reduction of natural protein intake to 1 g/kg/day and commencement of essential amino acid supplementation.

The patient underwent liver transplantation at age 17. There were some initial postoperative challenges, including a bile leak; however, he made a steady recovery and was discharged home 35 days post‐transplant, with full dietary liberalisation. His cognition has not been formally assessed, but he has finished high school, attained employment and is able to hold a conversation. He is in a long‐term relationship and has fathered a male child.

Routine Sanger sequencing of the *OTC* gene, in 1998, failed to identify a disease‐causing variant.

## 5 IDENTIFICATION OF THE MOLECULAR BASIS OF OTCD

**FIGURE 1:**
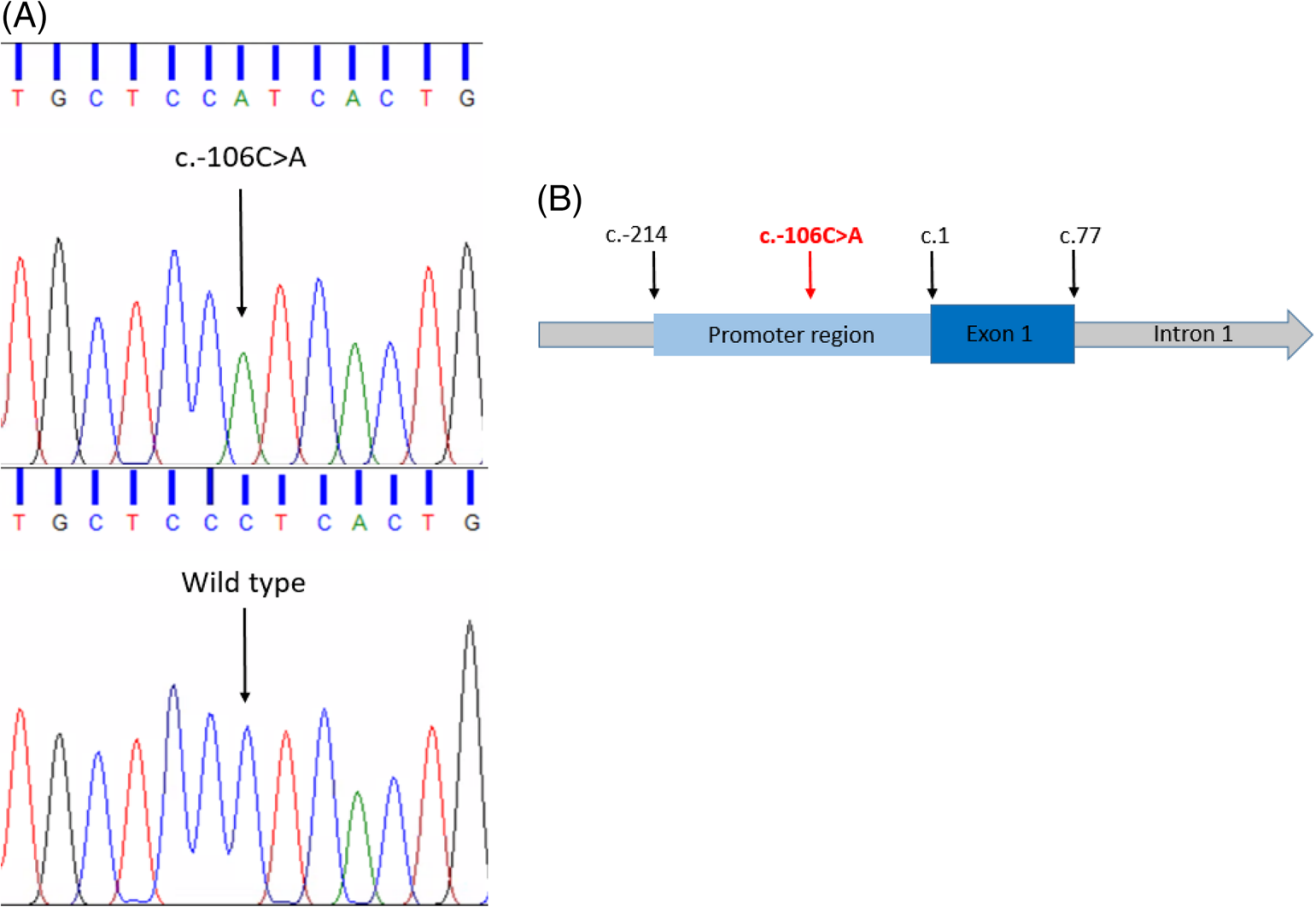
Genomic position of the c.‐106C>A variant in the *OTC* gene. (A) Sanger sequencing traces of the c.‐106C>A hemizygous variant identified in our three cases, compared to the wild‐type reference sequence. (B) Schematic representation of the promoter region of the *OTC* gene. The position of the start of translation is referenced by c.1 and the position of the c.‐106C>A variant is marked within the promoter of the 5′ untranslated region

All three patients were diagnosed biochemically with OTCD shortly after the onset of symptoms. However, a molecular aetiology proved elusive for these patients for almost two decades. Although initial Sanger sequencing of Patient 1 did not reveal a pathogenic variant, MLPA analysis identified a reproducible ~25% reduction in a single probe representative of *OTC* exon 1. This result indicated a possible mosaic deletion of this region, and a high‐resolution chromosome X microarray was used to explore this finding. No mosaic copy number change was detected within this region. Alternate primers were designed to encompass the region of the MLPA probe, which was situated in the upstream promoter region of *OTC*, in anticipation that this possible deletion was beyond the resolution of the microarray. Sanger sequencing of this promoter region identified a single nucleotide substitution, c.‐106C > A (NC_000023.10:g.38211844C>A; Figure 1). As this variant lies within the MLPA probe binding site, probe binding efficiency was affected, leading to the artefactual MLPA result seen in this patient.

A cohort of other male patients referred to our facility for sequencing of the *OTC* gene, without a molecular aetiology for their OTC deficiency, were re‐sequenced with these primers, thus identifying this recurrent variant in Patients 2 and 3.

Subsequent segregation studies identified two female heterozygotes (sister and mother of Patient 3), the latter of whom had positive protein and allopurinol challenge tests. The mother of Patient 1 and the maternal grandmother of Patient 3 also had similar challenge test results, without molecular confirmation. However, none of these females have had any metabolic decompensations to‐date.

## 6 DISCUSSION

Approximately 80%–90% of patients with biochemically‐confirmed OTC deficiency have a disease‐causing variant identified,^5^ largely within the coding region or intron/exon boundaries of the *OTC* gene. However, it is increasingly recognised that there are other causative variants in the noncoding regions of the human genome, and this is also true for OTCD.

Ogino and colleagues^7^ reported a case that presented with hyperammonaemic encephalopathy (ammonia >230 μmol/L) in the first day of life. OTC enzyme activity in the liver was undetectable. Initial Sanger sequencing of the *OTC* gene did not identify a causative variant. Subsequently, analysis of mRNA from the patient's liver, identified an insertion of a 135‐nucleotide sequence between exons 5 and 6, caused by a deep intronic single nucleotide substitution, c.540+265G>A, which activated a cryptic splice site within intron five.^7^ Additionally, Kumar and colleagues^8^ identified the same variant in three out of seven patients who had no pathogenic variants identified by previous molecular investigations.

Other mechanisms have also been associated with milder phenotypes in males. A frameshift variant identified in a male presenting at 18 months of age with vomiting, coma and hyperammonaemia was subsequently identified to be mosaic (60% in lymphocytes, 65% in oral mucosa).^10^ In this case, a null variant predicted to cause severe neonatal‐onset disease, was ameliorated by the residual wild‐type allele.

Jang and colleagues^9^ provide the only report to‐date of promoter variants causing OTCD. In this study, they sequenced the conserved regulatory regions (including the promoter, and an enhancer region nine kilobases upstream) of nine males from different families with clinical or biochemical features suggestive of OTCD. There was marked phenotypic variability among these patients (ranging from neonatal presentation to asymptomatic); however, two‐thirds were late‐onset. Five different promoter region variants, and a single enhancer region variant, were identified. All variants were functionally validated using either luciferase reporter or DNA pull‐down assays.^9^ Three patients harboured the same c.‐106C>A variant discussed here, suggesting that this may be a recurrent variant in males with late‐onset OTCD.

Our case series adds to the limited literature on promoter variants in association with late‐onset OTCD. None of the patients had neonatal‐onset (before 4 weeks of age) disease, with only mild ammonia elevations (if at all) at the time of biochemical diagnosis. However, there are also notable differences in their disease progression. Patient 3 experienced substantial morbidity associated with metabolic decompensations, with 87 hospital admissions (within 12 years), and required a liver transplant to preserve his cognition; it is possible that poor compliance with management strategies may have contributed to this. Patient 2 had self‐selected a low‐protein diet and had only three decompensations requiring hospital admissions; he is maintained on a tight protein restriction with average cognition. Patient 1 is on a similar protein restriction and has never had a metabolic decompensation. Despite the differences in their clinical journeys, all three patients have far better outcomes than those of males with the neonatal‐onset form of the disease.

It is important to note that, historically, molecular analysis of the *OTC* gene did not often cover deep intronic or the promoter regions. Our case series is part of an increasing body of literature reporting these molecular aetiologies. Targeted screening of these additional regions of the *OTC* gene should be considered in any patient with a clear biochemical diagnosis of OTCD, with no causative variants identified on routine molecular analysis. With the cost of whole‐genome sequencing rapidly declining, coverage of these regions will become easier and more cost‐effective, helping to maximise diagnostic yield.

## CONFLICT OF INTEREST

Ashley Hertzog, Arthavan Selvanathan, Rebecca Halligan, Timothy Fazio, Gerard de Jong, Drago Bratkovic, Kaustuv Bhattacharya, Adviye Ayper Tolun, Bruce Bennetts and Katrina Fisk declare that they have no conflicts of interest. This manuscript has not been submitted for review elsewhere and has been approved by all other authors for submission.

## ANIMAL RIGHTS

This article does not contain any studies with animal subjects performed by any of the authors.

## ETHICS STATEMENT

All procedures followed were in accordance with the ethical standards of the responsible committee on human experimentation (institutional and national) and with the Declaration of Helsinki of 1975, as revised in 2000. This project has received approval from the local human research ethics committee (CCR2021/03) and signed consent was obtained.

## Data Availability

The data from this case series is confidential patient information and, as such, is not publically available. However, the authors are contactable regarding case specifics.
